# Redox regulation of tumor suppressor PTEN in cell signaling

**DOI:** 10.1016/j.redox.2020.101553

**Published:** 2020-05-03

**Authors:** Ying Zhang, Jiyoung Park, Seong-Jeong Han, Sung Yeul Yang, Hyun Joong Yoon, Iha Park, Hyun Ae Woo, Seung-Rock Lee

**Affiliations:** a Department of Biochemistry, Department of Biomedical Sciences, Research Center for Aging and Geriatrics, Research Institute of Medical Sciences, Chonnam National University Medical School, Gwangju, 501-190, Republic of Korea; b College of Pharmacy, Graduate School of Pharmaceutical Sciences, Ewha Womans University, Seoul, 120-750, Republic of Korea; c COTDE Inc. 19-3, Ugakgol-gil, Susin-myeon, Cheonan-si, Chungcheongnam-do, 330-882, Republic of Korea

**Keywords:** PTEN, Redox regulation, Peroxides, Trx dimerization, Prx dimerization, Prx hyperoxidation

## Abstract

Phosphatase and tensin homologs deleted on chromosome 10 (PTEN) is a potent tumor suppressor and often dysregulated in cancers. Cellular PTEN activity is restrained by the oxidation of active-site cysteine by reactive oxygen species (ROS). Recovery of its enzymatic activity predominantly depends on the availability of cellular thioredoxin (Trx) and peroxiredoxins (Prx), both are important players in cell signaling. Trx and Prx undergo redox-dependent conformational changes through the oxidation of cysteine residues at their active sites. Their dynamics are essential for protein functionality and regulation. In this review, we summarized the recent advances regarding the redox regulation of PTEN, with a specific focus on our current state-of-the-art understanding of the redox regulation of PTEN. We also proposed a tight association of the redox regulation of PTEN with Trx dimerization and Prx hyperoxidation, providing guidance for the identification of novel therapeutic targets.

## 1 Introduction

Reactive oxygen species (ROS) are inevitably generated during aerobic and anaerobic metabolism and have detrimental effects on cellular biomolecules under pathological conditions [1]. Increasing evidence has indicated that ROS, such as H_2_O_2_, are produced and employed in physiological settings to serve as important signaling messengers for coordinating a variety of physiological functions, including proliferation, differentiation, apoptosis, signal transduction, and other critical events [[2], [3], [4], [5]]. To function as signaling messengers, these reactive molecules mainly trigger reversible oxidative post-translational modifications (PTMs) of reactive cysteine residues in regulatory proteins [6]. Because of the lack of enzymes to remove hydroxyl radicals and reactive aldehydes, their aggressive reactivity leads to the irreversible oxidation or degradation of functional proteins [7,8], a mechanism underlying various disorders and pathologies, such as diabetes, obesity, and cancer [9,10].

Signaling through PI3K/AKT is pivotal to cell growth and survival. The interaction of growth factors with receptor tyrosine kinases (RTK) typically activates PI3K. It has been shown that external stimuli, such as insulin, cytokines, neurotransmitters, peptide growth factors, and hormones, can activate the PI3K pathway, which results in ROS generation [5,[11], [12], [13]]. Upon the activation of cells by growth stimuli, classic PI3K family members catalyze the phosphorylation of phosphatidylinositol 4,5-bisphosphate (PIP2) to phosphatidylinositol 3,4,5-trisphosphate (PIP3), a potent activator of 3-phosphoinositide-dependent kinase (PDK) and AKT. PTEN is a nonredundant, plasma-membrane lipid phosphatase that can antagonize PI3K by dephosphorylating PIP3 at position D3 to generate PIP2. Numerous studies have demonstrated that the catalytic activity of PTEN is modulated by ROS, subsequently resulting in its catalytic inhibition [14,15]. Therefore, the oxidative modification of PTEN contributes to augmenting PIP3 levels, resulting in the activation of the PI3K/AKT signaling pathway and protecting cells from oxidative stress-induced cell death.

Endogenous antioxidants, such as thioredoxins (Trxs) and peroxiredoxins (Prxs), can modulate ROS levels and intracellular redox state. Thus, they have become targets for redox modifications [16]. Trxs can catalyze the reversible reduction of protein disulfide bonds using redox-active cysteine residues in their active site. Prxs are a family of thiol-dependent antioxidants that can scavenge cytosolic or mitochondrial peroxides. Trx and Prx undergo redox-dependent modifications of catalytic amino acids that can affect protein functionality or impact subcellular protein targeting, protein-protein interactions, or protein stability. In this review, we focused on recent achievements and novel insights into the redox regulation of PTEN induced by peroxides. We also discussed the tight connection of PTEN redox regulation with Trx dimerization and Prx hyperoxidation.

## 2 Cellular functions of PTEN

**Fig. 1:**
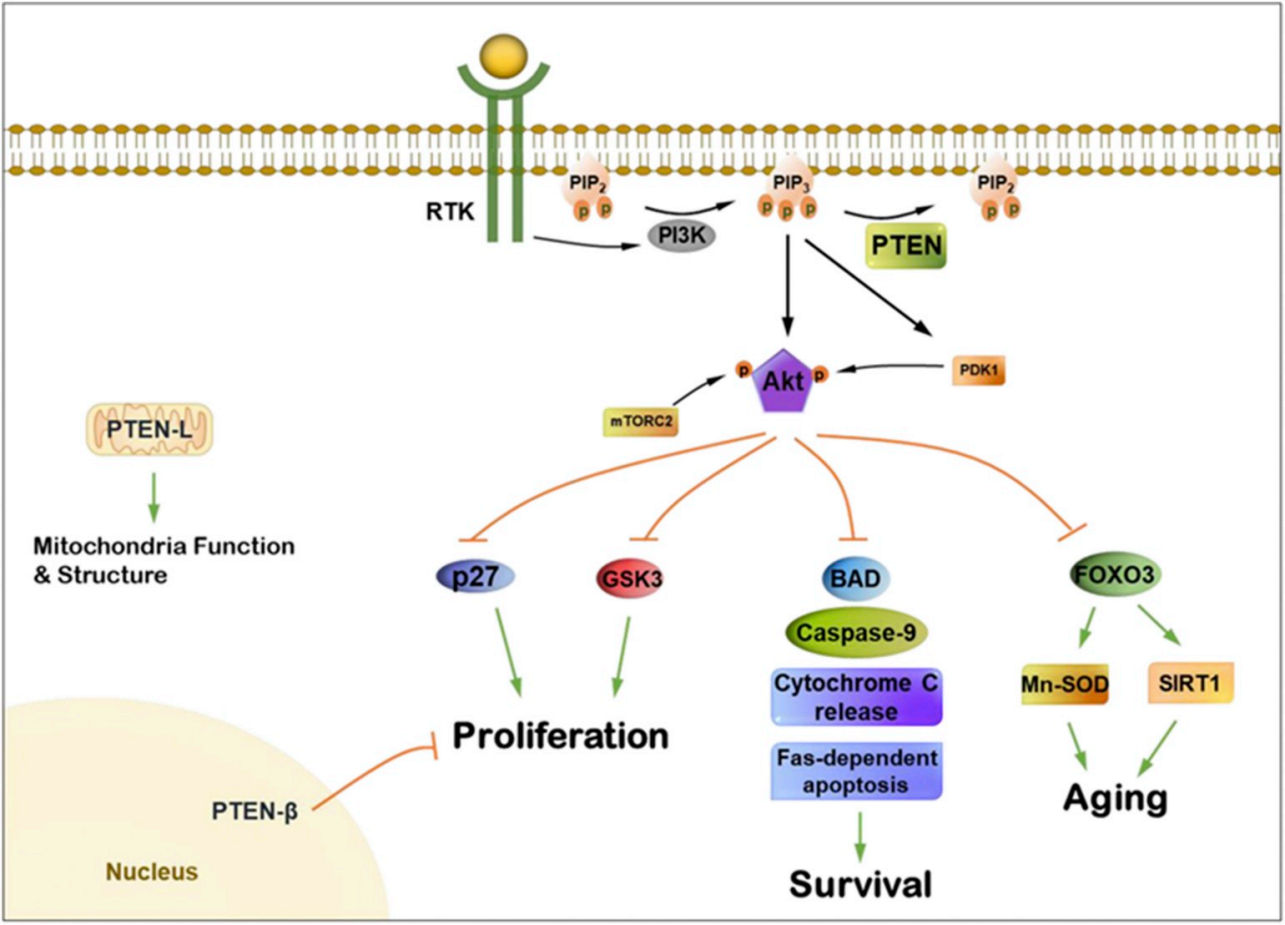
Role of PTEN in the control of cell signaling. PTEN coordinates cell growth, survival, proliferation, and metabolism by opposing activation of the PI3K/Akt signaling pathway. Two isoforms of canonical PTEN have been identified, PTEN-Long (PTEN-L) and PTEN-β. PTEN- L is a membrane-permeable lipid phosphatase that is secreted from cells and can be taken up by other cells directly. PTEN-L is also localized in the mitochondrial and can regulate mitochondrial functions and energy production by associating with canonical PTEN to increase PTEN-induced putative kinase 1 (PINK1) expression. PTEN-β localized in the nucleolus negatively regulates ribosomal DNA transcription and ribosomal biogenesis by physically interacting with and dephosphorylating nucleolin. Canonical PTEN can also be secreted and taken up by other cells, which together with PTEN-L secretion, contributes to the non-cell autonomous effects of PTEN [105].

PTEN, a redox-sensitive dual-specificity protein and lipid phosphatase, was first identified as a tumor suppressor. It is frequently lost in a variety of human tumors of numerous tissues including the brain, breast, and prostate [17]. PTEN is one of the most frequently mutated tumor suppressor genes in human cancers. Even a subtle decrease in PTEN level and/or activity can result in cancer susceptibility and favor tumor progression. The crystal structure of human PTEN protein has revealed an N-terminal PIP2-binding/phosphatase domain (PBD), a lipid-binding C2 domain, a C-terminal tail domain (C-tail), and a class I PDZ (PSD-95, DLG1, and ZO-1) binding domain. PTEN is known as a lipid phosphatase for PIP3. PIP3 functions as a secondary messenger that regulates cell polarity and migration [18]. PTEN can then coordinate cell proliferation, growth, survival, and metabolism by negatively regulating the PI3K/AKT/mTOR signaling pathway [[19], [20], [21]]. Additionally, it has been reported that PTEN can directly dephosphorylate residues on itself and several other protein substrates, including phosphoprotein focal adhesion kinase 1 (FAK), Shc, cAMP-responsive element-binding protein 1 (CREB1), insulin receptor substrate 1 IRS1, Dishevelled (DVL), and others to exert its tumor suppressive functions [[22], [23], [24], [25], [26], [27]]. Molecular details regarding PTEN in the regulation of cellular signaling pathways (Fig. 1) and its role in human diseases have been described in several reviews [[28], [29], [30]].

A growing body of evidence has indicated that PTEN can exert part of its tumor suppressive functions by controlling cell migration, spreading, focal adhesions, and genomic stability, independent of its phosphatase activities [23,28,31]. For instance, microspherule protein 1 (MSP58)-mediated cellular transformation is inhibited by its physical interaction with the C-terminal domain of PTEN [32]. A recent study showed that cytosolic PTEN could suppress CHD-1 induced trimethyl lysine-4 histone H3 modification by stimulating chromodomain-helicase-DNA-binding protein 1 (CHD1) proteasomal degradation, leading to the transcriptional activation of the TNF/NF-κB pathway [33]. Accordingly, *in vivo* genetic analyses in mouse models have revealed that PTEN loss is not synonymous with AKT overexpression [34].

Recent studies revealed the existence of translational isoforms of PTEN, PTEN-Long (PTEN-L) and PTEN-β. Like canonical PTEN, PTEN-L is a membrane-permeable lipid phosphatase that is secreted from cells and can be taken up by other cells directly. PTEN-L, also localized in the mitochondria, can regulate mitochondrial functions and energy production by associating with canonical PTEN to increase PTEN-induced putative kinase 1 (PINK1) expression [35,36]. PTEN-β localizes in the nucleolus and negatively regulates ribosomal DNA transcription and ribosomal biogenesis by physically interacting with and dephosphorylating nucleolin [37]. With a high sequence homology to canonical PTEN, PTEN-L, and PTEN-β may be modulated by the same or similar mechanisms.

Disruptions in the regulation of PTEN by a range of molecular mechanisms can generate various dysfunctional PTEN species and/or a spectrum of PTEN levels that can variously contribute to the pathogenesis of inherited syndromes, including Cowden disease, Lhermitte-Duclos syndrome, Bannayan-Zonana Syndrome [38], cancers, and other diseases. These molecular mechanisms include the epigenetic loss and mutation of PTEN; transcriptional regulations; post-transcriptional regulation, including microRNA, the disruption of competitive endogenous RNA (ceRNA) networks, and long non-coding RNAs; post-translational modifications; and the aberrant localization of PTEN. PTEN function is also finely regulated through protein-protein interactions [28,39]. More recently, evidence has shown that PTEN is capable of forming dimers, which has been proven to be a novel mechanism for its functional regulation [40]. The following sections highlight our current understanding of the redox regulation of PTEN in cell biology.

## 3 Redox regulation of PTEN by peroxides, thioredoxin, and peroxiredoxin

### 3.1 Oxidative inactivation of PTEN by H_2_O_2_

**Fig. 2:**
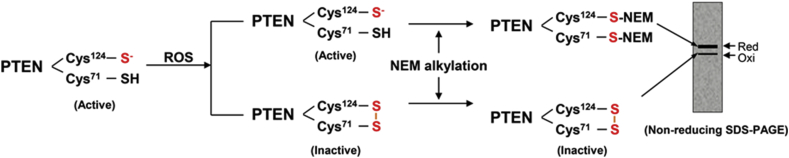
Experimental scheme for monitoring the redox status of PTEN by mobility shift. N-ethylmaleimide (NEM) reacts with reduced PTEN thiols resulting in PTEN-NEM adducts, which increase the protein mass. This additional molecular weight leads to a slower shift on non-reducing SDS-PAGE, generating an additional upper band on the gel. In contrast, if PTEN thiols are oxidized to form a disulfide bond, they will not react with NEM and mobility shift will be observed. This approach allows determination of the ratio between the oxidized (intra-PTEN disulfide, inactive) and reduced (PTEN-red, active) forms of PTEN after exposure to oxidants.

**Fig. 3:**
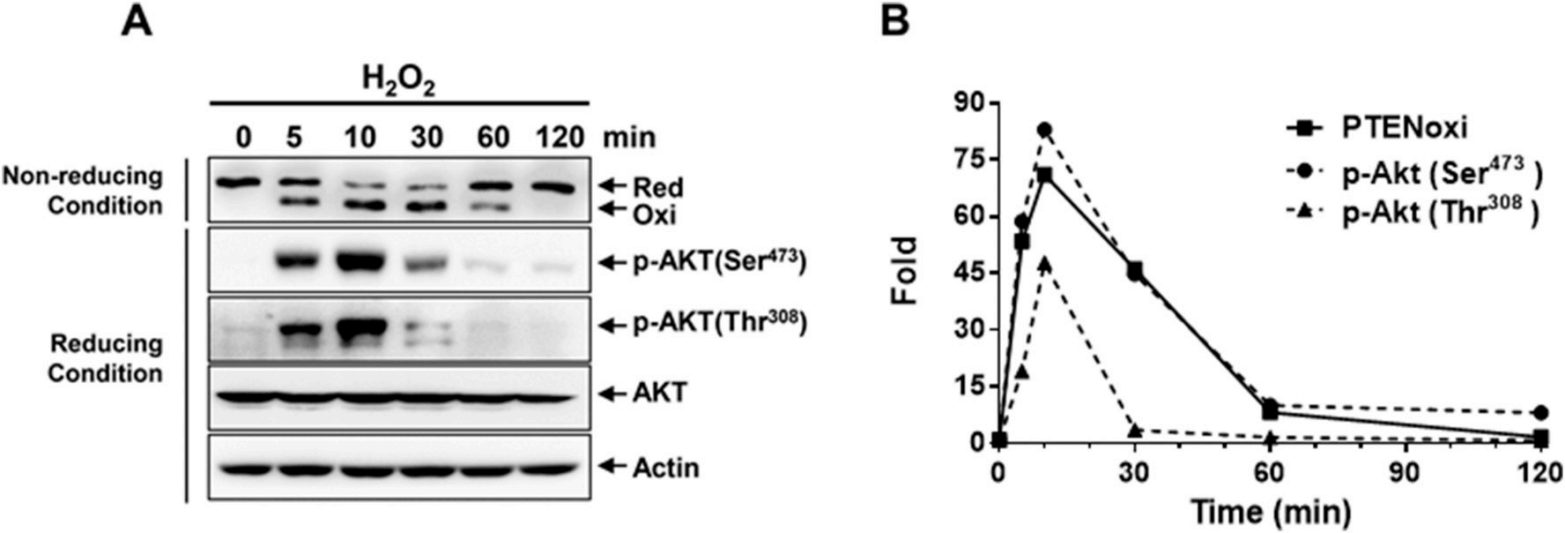
H_2_O_2_-induced PTEN reversible oxidation and AKT phosphorylation in HeLa cells. (A) Serum-starved HeLa cells were exposed to 1 mM H_2_O_2_ for various times. All samples were alkylated with 10 mM NEM and subjected to non-reducing or reducing SDS-PAGE, followed by immunoblot analysis with PTEN, *p*-AKT (Ser^473^), *p*-AKT (Thr^308^), total AKT or actin antibodies. (B) The intensity of the oxidized PTEN bands and *p*-AKT bands from (A) was quantitated using ImageJ software. Red, reduced PTEN; Oxi, oxidized PTEN.

**Fig. 4:**
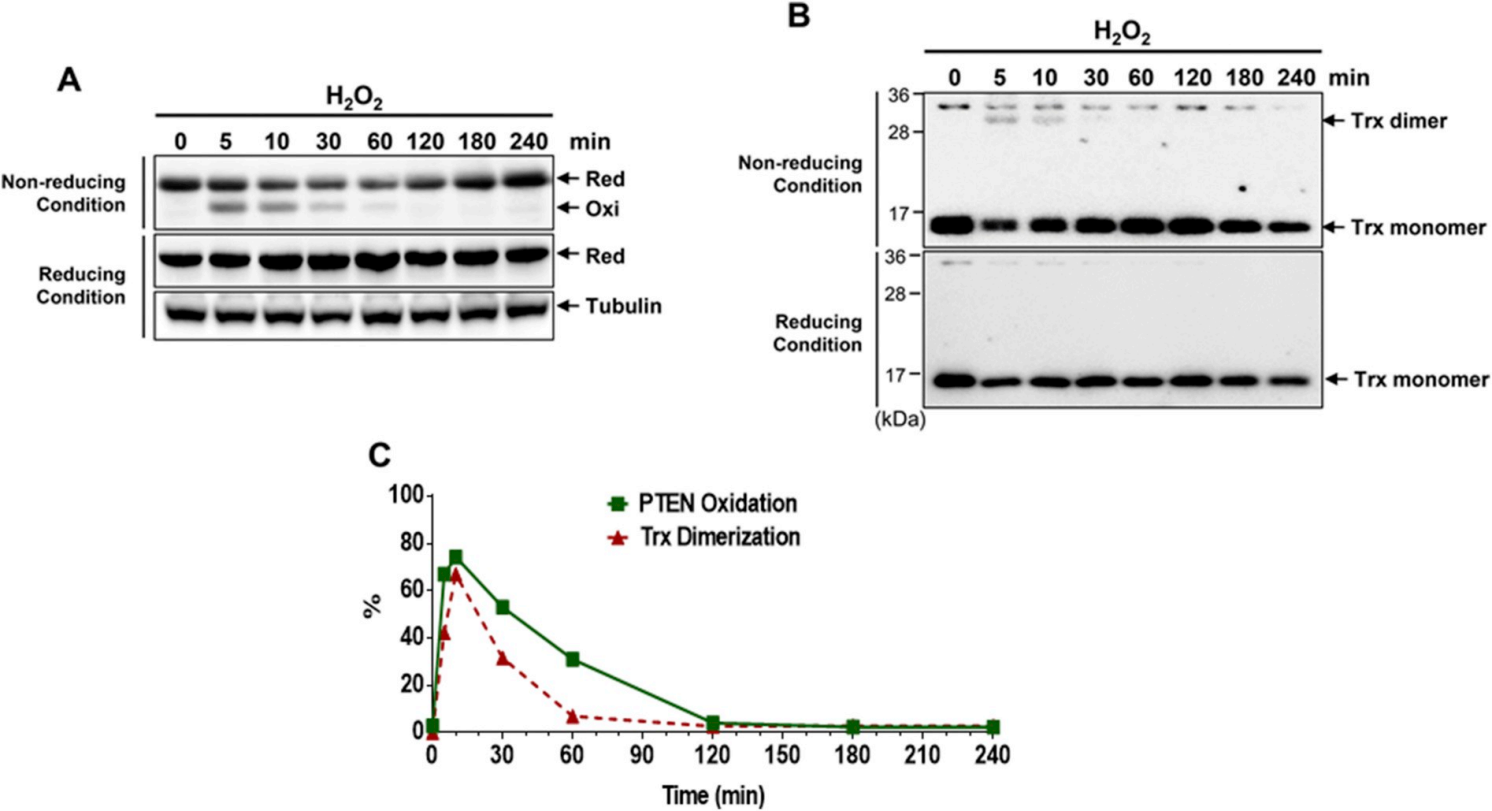
H_2_O_2_-induced Trx dimerization in HeLa cells. (A–B) Serum-starved HeLa cells were exposed to 1 mM H_2_O_2_ for various times. All samples were alkylated with 10 mM NEM and subjected to non-reducing or reducing SDS-PAGE, followed by immunoblot analysis with PTEN, Trx or tubulin antibodies. (C) The intensity of the oxidized PTEN bands from (A) and the Trx dimer bands from (B) was quantitated using ImageJ software. Red, reduced PTEN; Oxi, oxidized PTEN

PTEN is sensitive to oxidation because it contains nucleophilic cysteine residues in the active site. It has been reported that the catalytic activity of PTEN is fine-tuned by the exposure to oxidizing agents *in vitro* or oxidative stress conditions in cells. The essential active Cys^124^ residue of human PTEN, surrounded by three basic amino acid residues in the active site pocket, is readily oxidized by forming an intramolecular disulfide with Cys^71^ [15], resulting in the inhibition of its phosphatase activity. Importantly, H_2_O_2_-mediated PTEN oxidation is reversible, which is predominantly reduced by thioredoxin. Conversely, cellular PTEN activity can be protected by the presence of ROS scavengers [41]. We have designed a convenient approach to monitor intra-PTEN disulfide using a mobility shift assay [15,42] (Fig. 2). In this procedure, all free cellular thiols and selenols of proteins are first blocked by alkylation with N-ethylmaleimide (NEM) and the alkylated PTEN confers a higher molecular weight. The proteins were then separated under non-reducing conditions in the presence of sodium dodecyl sulfate (SDS) and subjected to immunoblots using antibodies to PTEN. Differences in molecular weight and conformational structure, the oxidized and reduced forms of PTEN can then be visualized [15]. Treatment of HeLa cells with H_2_O_2_ resulted in PTEN oxidation in a time-dependent manner, with the maximal oxidation after 10 min of exposure. The oxidized PTEN was then converted to the reduced form, mostly after 120 min of exposure (Fig. 3). This clearly indicates that the oxidation reaction in the cells was reversible. In cells exposed to H_2_O_2_, the augmented oxidation of PTEN was accompanied by an elevation in the amount of cellular PIP3 [15] and the downstream activation of AKT through its phosphorylation, both on Ser^473^ (pAkt^Ser473^) and Thr^308^ (pAkt^Thr308^) (Figs. 3 and 4A). Phosphorylation of both Thr^308^ and Ser^473^ residues is required for the full activation of Akt. H_2_O_2_-dependent increases in PIP3 abundance and Akt activity were correlated with the oxidative inactivation of PTEN in PTEN-expressing glioma cells, but not in PTEN-null glioma cells, further supporting the notion that oxidative stress regulates PI3K-dependent signaling through the oxidative inhibition of PTEN [[43], [44], [45]].

***Catalytic activity of PTEN is modulated by thioredoxin****.* Oxidation-driven PTEN inactivation can be reversed by cellular reducing systems, among which the thioredoxin (Trx) system plays a major role. Trx plays an important role in PTEN activation after H_2_O_2_ treatment. It has been demonstrated that Trx is even more efficient than glutathione or glutaredoxin in the reduction of oxidized PTEN [15]. Trx is a ubiquitous protein presents in all species from archaebacteria to human. Functions of Trx have been subjected in many investigations [46]. Primarily, the reduced form of Trx serves as a general protein thiol-disulfide oxidoreductase. The evolutionarily conserved Trp-Cys^32^-Gly-Pro-Cys^35^ catalytic center provides sulfhydryls involved in Trx-dependent reducing activity. The N-terminal cysteine of the reduced form Trx initiates a nucleophilic attack on the target protein disulfide and forms a transient mixed disulfide which is subsequently attacked by the C-terminal cysteine of Trx to generate the reduced target protein and oxidized Trx with a disulfide bond. Such oxidized Trx is recycled by Trx reductase (TrxR), which utilizes NADPH as an electron donor [47]. Thus, the Trx system is comprised of Trx, selenoenzyme TrxR, and NADPH [48,49], known to have pivotal roles in the regulation of redox signaling via maintaining the thiol-related redox status balance.

Previous studies have revealed that *E. coli* and human Trxs act as monomers [[50], [51], [52]]. It has been observed that Trxs derived from vertebrates are more susceptible to oxidation that forms dimers or oligomers via Cys residues [50]. Except for the formation of an active Cys^32^-Cys^35^ disulfide, a non-active Cys^62^-Cys^69^ disulfide bond, dimers, and multimers are also formed under long exposure to air or high levels of H_2_O_2_ and diamide [50,51,53,54]. Crystallographic studies have revealed that dimerization through an intermolecular disulfide bond between Cys^73^ and Cys^73’^ of two monomers renders Trx unable to carry out its redox activity compared to the crystallized Trx-TrxR complex [50,55]. The homodimerization of Trx started to accumulate 5 min after H_2_O_2_ treatment and lasted until 60 min of incubation in HeLa extracts (Fig. 4B). However, the Trx dimer disappeared after 120 min of incubation, showing a similar trend in the reduction kinetics of H_2_O_2_-oxidized PTEN. This suggests the H_2_O_2_-induced production of Trx dimers in response to elevated levels of oxidized PTEN. Dimeric Trx has long been considered as a potential cellular regulatory redox signaling molecule that might be a possible target for the development of anticancer drugs [56]. Our studies have reported that cellular Trx is one of the targets of organic hydroperoxides that can induce Trx dimerization and oligomerization, causing the irreversible oxidation of PTEN [49,57].

Trx is overexpressed in many human cancers. It is associated with increased tumor cell proliferation, inhibited apoptosis, and decreased patient survival [58,59]. It has been previously reported that increased levels of Trx1 can bind to PTEN in a redox-dependent manner to inhibit its PtdIns-3-phosphatase activity, resulting in increased Akt activation in cells [60]. The interaction between Trx-1 and PTEN occurs through a disulfide bond between the active site Cys^32^ of Trx-1 and Cys^212^ of PTEN, inhibiting PTEN's lipid phosphatase activity and increasing tumorigenesis [60]. This provides an additional mechanism for tumorigenesis with a loss of PTEN activity. However, the inactivation of PTEN by Trx can be reversed in the presence of thioredoxin-interacting protein (Txnip). Upon Txnip interaction with Trx through a disulfide bond between Cys^247^ of Txnip and C^32^ of Trx, Trx is no longer able to bind and allow for reactivate PTEN [61].

***Catalytic activity of PTEN is modulated by peroxiredoxin.*** Peroxiredoxins (Prxs) are a ubiquitously expressed family of small non‐seleno peroxidases (22–27 kDa) that can catalyze reduction of H_2_O_2_, organic hydroperoxides, and peroxynitrite using reducing equivalents provided by thiol-containing proteins [62]. Mammalian cells possess six isoforms of Prxs. They can be classified into three subgroups: four 2-Cys Prx isoforms (Prx1-4), one atypical 2-Cys Prx isoform (Prx5), and one 1-Cys Prx isoform (Prx6) [62]. Although these six mammalian Prx isoforms have different individual functions in cellular redox regulation and antioxidant protection, they all enable to regulate intracellular H_2_O_2_ levels by catalyzing peroxide reduction for signaling and metabolism [63]. All mammalian Prx enzymes are homodimers arranged in a head-to-tail orientation. They contain a conserved cysteine residue in the N-terminal region that is the primary site of the oxidation of H_2_O_2_. In the catalytic cycle of 2-Cys Prx proteins, the conserved C_P_-SH (Cys^51^ in Prx1) is selectively oxidized to C_P_-SOH intermediate at low levels of H_2_O_2_. After reacting with the C-terminal-conserved C_R_-SH (Cys^172^ in Prx1) of the other subunit in the homodimer, an intermolecular disulfide is generated. Ultimately, it is specifically reduced by Trx [62,64]. Under normal cellular homeostasis with low H_2_O_2_, rather than displaying peroxidase activity, predominantly low-molecular weight oligomeric 2-Cys Prxs can also protect proteins from degradation. With higher doses of H_2_O_2_, C_P_-SOH generated as an intermediate during catalysis, occasionally undergoes further oxidation to C_P_-SO_2_H catalysis in the presence of Trx, resulting in the inactivation of its peroxidase activity. The generated C_P_-SO_2_H can be reduced back to thiol by sulfiredoxin (Srx) [65,66]. However, further oxidation to C_P_-SO_3_H is irreversible, resulting in Prx degradation [65]. Recently, it has been reported that when Cys^51^ in Prx1 was in an overoxidized form due to oxidative stress or heat shock stress, the formation of high molecular weight complexes was favored [67]. The structural change from low molecular weight oligomers to high molecular weight complexes is accompanied by a functional change from peroxidase to molecular chaperone activity [68,69]. The peroxidase function of Prx can be regulated by various post-translational modifications, including phosphorylation, lysine acetylation, glutathionylation, nitrosylation, and thiol oxidation [[70], [71], [72], [73], [74]].

**Fig. 5:**
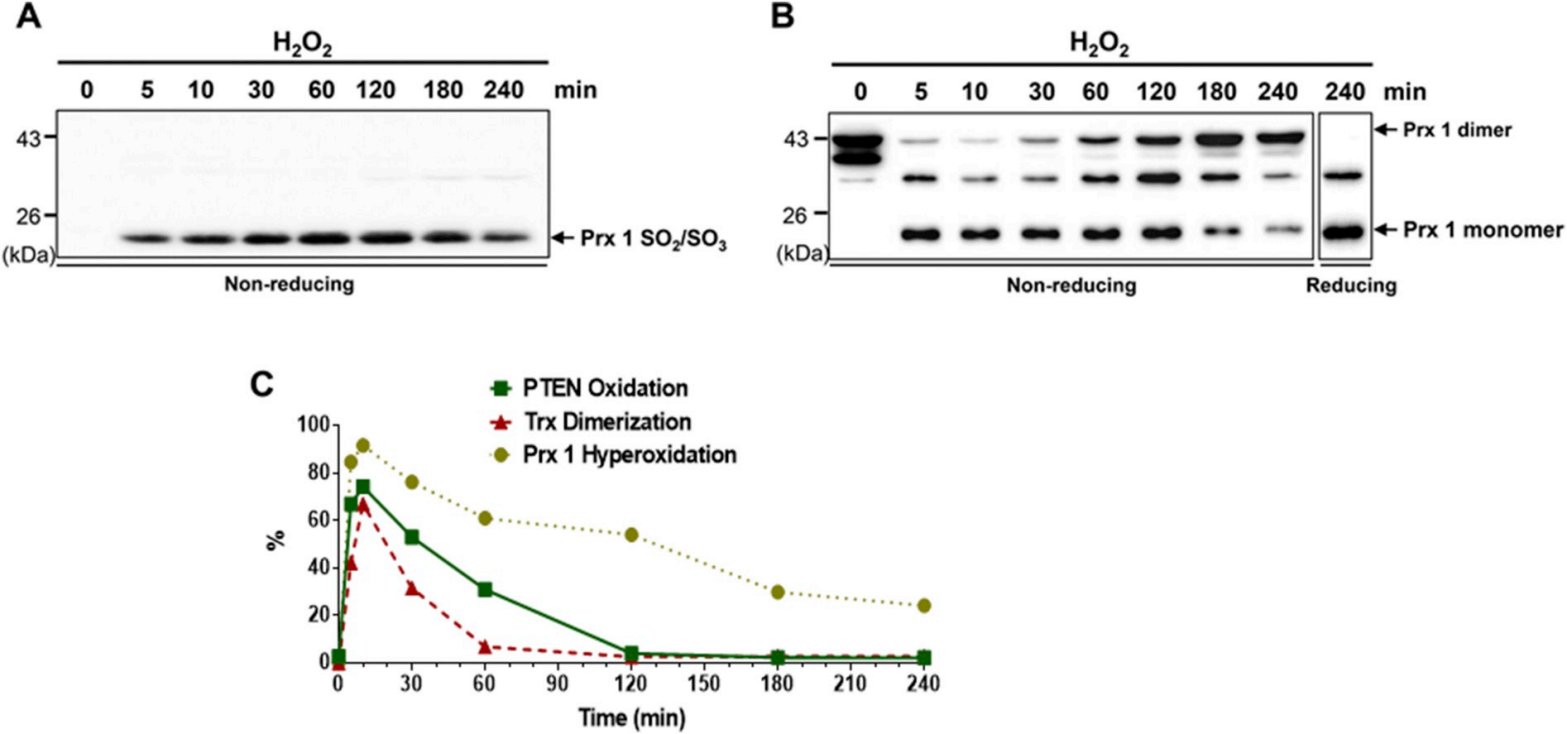
H_2_O_2_-induced Prx hyperoxidation in HeLa cells. (A–B) Serum-starved HeLa cells were exposed to 1 mM H_2_O_2_ for various times. Cells were lysated without NEM and subjected to non-reducing or reducing SDS-PAGE, followed by immunoblot analysis with Prx 1 SO_2_/SO_3_ or Prx 1 antibodies. (C) The intensity of the oxidized PTEN bands from (Fig. 3A) and the Prx hyperoxidation bands from (B) was quantitated using ImageJ software. Monomers represent the hyperoxidized species only, whereas dimers represent the sum of the reduced and dimeric proteins at the time of lysis. Red, reduced PTEN; Oxi, oxidized PTEN

It has been reported that Prx1 can protect and promote PTEN tumor suppressive function by interacting with PTEN and protecting disulfide bond formation under mild oxidative stress [75]. The lipid phosphatase activity of PTEN was fully preserved by Prx1 in cells under low concentrations of H_2_O_2_ (25 μM), where Prx1 was found to interact with PTEN. However, under higher concentrations of H_2_O_2_ (500 μM), Prx1 was irreversibly hyperoxidized and dissociated from PTEN [75]. Prx1 preserved PTEN lipid phosphatase activity under oxidative stress at a 1:1 (mol:mol) ratio of Prx1 and PTEN, and could not be further enhanced by excess Prx1, indicating that Prx1 most likely interacts with PTEN as a monomer [75]. Mutational analysis and computational analysis suggested that Prx1 interacts within the C2 domain of PTEN (aa 186–274) and PTEN with the N-terminal of Prx1 (aa 1–21) and the C- terminal of Prx1 (aa 183–199) [75]. Hyperoxidized Prx was observed when cells were exposed to H_2_O_2_ for various times using an antibody specific against cysteine sulfinic acids (Fig. 5A). The basal Prx1 dimer was readily observed in H_2_O_2_-untreated HeLa cells because Prxs are predominantly obligate dimers at the time of lysis in the absence of NEM. Prx1 hyperoxidation was increased maximally after 5 min of exposure and then decreased progressively over time (Fig. 5B). In addition, augmentation with oxidized PTEN induced by H_2_O_2_ showed kinetics similar to H_2_O_2_-induced Prx1 hyperoxidation (Fig. 5C). The temporary inhibition of Prx by hyperoxidation impaired H_2_O_2_-scavenging activity.

Inactivated Trx dimerization resulted in a delay in the reduction of PTEN and Prx1. However, whether Prx1 reduced the H_2_O_2_-induced intramolecular disulfide bond in PTEN is still unclear. Consequently, the redox regulation of PTEN by H_2_O_2_ is mediated through Trx and Prx systems in cell signaling.

### 3.2 Oxidative inactivation of PTEN by organic hydroperoxides

Organic peroxides and hydroperoxides are tumor promoters [76]. The tumor promoting activity of organic hydroperoxides involves in generation of free radical derivatives [[77], [78], [79]]. Cumene hydroperoxide (CuHP) is a stable organic hydroperoxide with oxidizing capabilities [80]. CuHP can induce lipid peroxidation by reacting with adjacent fatty acid side-chains in the presence of transition metal, resulting in continuous generation of lipid hydroperoxides via chain reactions [81,82]. These lipid hydroperoxides, in turn, can generate several ROS, such as alkoxyl and peroxyl radicals *in vitro* and *in vivo*, thereby exerting various types of oxidative damage, including lipid peroxidation, protein modification, and DNA damage [80,83,84]. *Tert*-butyl hydroperoxide (*t*-BHP) is extensively metabolized in target issues to form several free radical intermediates, including phenoxyl, peroxyl, alkoxyl, and alkyl radical derivatives in murine keratinocytes [80], hemoglobin-thiyl and methyl radicals in rat liver stomach [85]. The cytotoxic effects of *t*-BHP are involved in glutathione depletion [86], hemoglobin oxidative denaturation, hemolysis and erythrocyte membrane lipid peroxidation [87,88], inner mitochondrial membrane permeabilization [89], DNA single strand breakage [90,91], and apoptosis [76]. Stimulation of HeLa cells or recombinant PTEN with either CuHP or *t*-BHP resulted in PTEN oxidation by forming an intramolecular disulfide between Cys^124^ and Cys^71^ in time- and concentration-dependent manners [49,57]. However, these organic hydroperoxides-mediated cellular PTEN oxidation was not reversible, because the cellular Trx was inactivated by dimerization [49,57]. In addition, the ablation of Prx enhanced CuHP-induced PTEN oxidation in MEF cells [49]. Overall, these results imply that PTEN is an important physiological target for organic peroxide-mediated redox signaling. Its irreversible oxidation could play a key role in organic peroxide-induced tumorigenesis.

### 3.3 Oxidative inactivation of PTEN by lipid peroxide

**Fig. 6:**
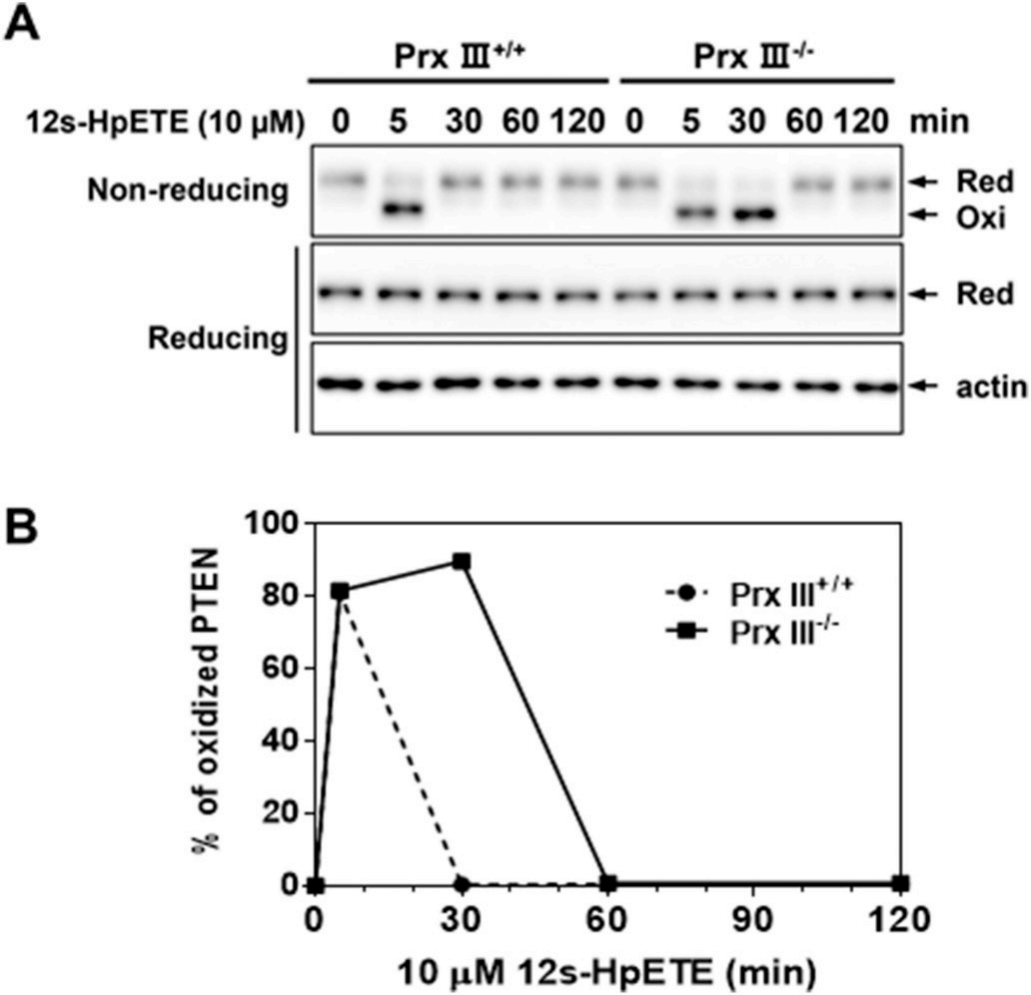
Effects of 12s-HpETE on the redox state of PTEN in Prx III^+/+^ and Prx III^−/-^ MEF cells. (A) MEF cells were treated with a mixture of 10 μM 12s-HpETE and Lipofectamine 2000 transfection reagent for the indicated times. The cellular protein extracts were alkylated with 10 mM NEM and subjected to nonreducing or reducing SDS-PAGE, followed by Western blot analysis using antibodies to PTEN or actin. (B) The intensity of oxidized PTEN was quantitated using ImageJ software.

Lipoxygenases (LOX) can catalyze the production of hydroperoxyeicosatetraenoic acid (HpETE) from arachidonic acid (AA). 15-LOX can metabolize AA to form 15(s)-hydroperoxyeicosatetraenoic acid (15s-HpETE), the oxidative precursor of 15-hydroxyeicosatetraenoic acid (15s-HETE). Human 15-LOX has two isoforms, 15-LOX-1 and 15-LOX-2. 15-LOX-1 is a dual-specificity enzyme that metabolizes AA, principally to 15s-HpETE, and to far smaller amounts of 12s-HpETE. 15-LOX-2 can metabolize AA to 15s-HpETE. It has little or no ability to metabolize AA to 12s-HpETE. These HpETEs are subsequently reduced and transformed to produce eicosanoids known to be important signaling molecules in immune responses and other physiological processes. Increased levels of lipid peroxides have been linked to the pathogenesis of a variety of human diseases, including neurodegeneration, atherosclerosis, type Ⅱ diabetes, metabolic disorders, solid tumors, and hematologic malignancies, through cellular oxidative damage [[92], [93], [94], [95]]. The ability of lipid peroxides to oxidize PTPs has been reported [96]. PTEN oxidation by unidentified AA metabolites has also been shown [97]. Both 15s-HpETE [98] and 12s-HpETE (Fig. 6) resulted in PTEN oxidation in MEF cells, and Prx III deletion aggravated the 12/15s-HpETE-induced PTEN oxidation in MEF cells. 15s-HpETE-mediated cellular PTEN oxidation was identical to that of H_2_O_2_, indicating that an intramolecular disulfide bond between Cys^124^ and Cys^71^ was formed [98]. However, 15s-HETE was unable to induce PTEN oxidation in MEF cells [98].

### *3.4* *S*-nitrosylation of PTEN by nitric oxide

PTEN is also modified by *S*-nitrosylation [[99], [100], [101]], a covalent modification of cysteine residues by nitric oxide (NO), which is another crucial redox mechanism that regulates PTEN activity. *S*-nitrosylation and H_2_O_2_-mediated oxidation occur on distinct Cys residues of PTEN. A recent study has shown thatNO can induce PTEN *S*-nitrosylation on Cys^83^, leading to the suppression of lipid phosphatase activity of PTEN and induction of PTEN protein degradation via the ubiquitin-proteasome system (UPS) through NEDD4-1-mediated ubiquitination [102,103]. Involvement of PARK2 encoding ubiquitin E3 ligase Parkin in PTEN *S*-nitrosylation has been reported [104]. Depletion of PARK2 enhanced the 5-AMP-activated protein kinase (AMPK)-mediated activation of endothelial nitric oxide synthase (eNOS), leading to increases in NO levels that drove *S*-nitrosylation and subsequent ubiquitination of PTEN [100]. These findings suggest that the *S*-nitrosylation of PTEN could serve as a possible new therapeutic target.

## 4 Concluding remarks

The reversible oxidation of Cys residues in proteins upon cellular oxidants is linked to signaling events. PTEN was discovered as a *bona fide* tumor suppressor. The importance of its functions in the regulation of cell growth, motility, and the inhibition of apoptosis has been well-established. ROS has been recognized as a secondary messenger that can modify cell signaling by oxidizing protein cysteine thiols. Challenges have moved from PTEN pleiotropic natural functions toward understanding its regulation. This review concentrated on the redox regulation of PTEN, which is crucially linked to its tumor suppressor function.

**Fig. 7:**
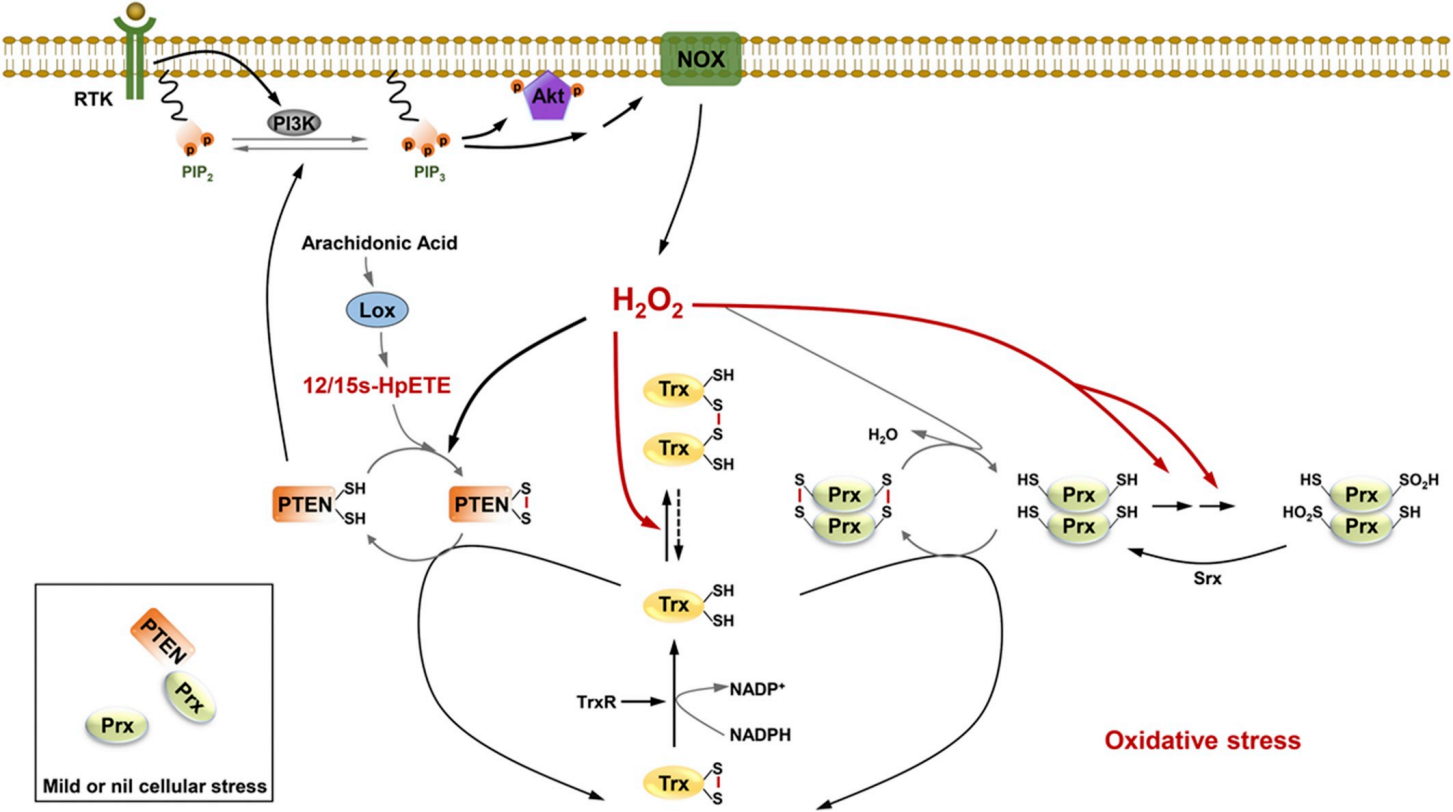
A schematic model of the effects of peroxides on the redox regulation of the tumor suppressor PTEN by the Trx system and Prx. Prx1 and PTEN interaction is essential for protecting PTEN from oxidation-induced inactivation. PTEN lipid phosphatase activity was fully protected by Prx1 in cells under low H_2_O_2_ exposure, where Prx1 was found to bind PTEN as a monomer [75]. H_2_O_2_ is scavenged by Prx1 in a controlled fashion, which itself becomes reversibly oxidized. However, under H_2_O_2_ treatment with higher concentrations, Prx1 is known to become irreversibly hyperoxidized and dissociate from PTEN. H_2_O_2_ can inactivate Prx in a two-step hyperoxidation of the active site Cys-SH to Cys-SO_2_H, which can be reactivated by Srx. Prx inactivation allows the accumulation of local H_2_O_2_, which in turn promotes the inactivation of PTEN by oxidizing its catalytic cysteine residues to an intradisulfide. In addition to the impaired activity of Prx, the intermolecular disulfide/dimerization of Trx also occurs, causing a loss of Trx reactivity, which in turn promotes the oxidation of PTEN. This inactivation of PTEN increases the abundance of phosphorylated AKT and sufficiently triggers downstream signaling events.

Trx and Prx play vital roles in the control of intracellular redox state of PTEN. The participation of H_2_O_2_ in intracellular signaling by targeting PTEN, Trx, and Prx, and the regulation of H_2_O_2_ concentrations by Prx are depicted schematically in Fig. 7. Growth factors induced the activation of PI3K, resulting in the conversion of PIP2 to PIP3. PIP3 induced the production of H_2_O_2_ by activating the NOX (Nicotinamide adenine dinucleotide phosphate oxidase) complex. The interaction of Prx1 and PTEN is essential for protecting PTEN from oxidation-induced inactivation. PTEN lipid phosphatase activity was fully protected by Prx1 in cells under low H_2_O_2_ exposure, where Prx1 was found to bind PTEN as a monomer [75]. The generated H_2_O_2_ mediated the inactivation of cytosolic Prx molecules located nearby through forming intermolecular disulfides. The disulfides were subsequently and specifically reduced by Trx, which in turn received reducing equivalents from NADPH via TrxR. However, when treated with higher concentrations of H_2_O_2_, Prx1 is known to become irreversibly hyperoxidized and dissociate from PTEN. H_2_O_2_ can inactivate Prx in a two-step hyperoxidation of the active site Cys-SH to Cys-SO_2_H, which can be reactivated via an ATP-dependent reduction catalyzed by sulfiredoxin (Srx). Prx inactivation allows the accumulation of local H_2_O_2_, which in turn promotes the inactivation of PTEN by forming a disulfide bond. In addition to impaired activity of Prx, the intermolecular disulfide/dimerization of Trx also occurs, causing a loss of Trx reactivity, which in turn, promotes the oxidation of PTEN. This inactivation of PTEN increases the abundance of phosphorylated AKT and sufficiently triggers downstream signaling events. The impaired activity of Trx caused by dimerization provides a mechanism by which Trx activity is transiently inhibited under the conditions of oxidative stress, providing more time for sensing and transmission of oxidative signals. However, further studies are needed to reveal the mechanisms that involved in Trx transient inhibition and the important roles of Trx and Prx in redox signaling closely associated with PTEN redox regulation.

## Declaration of competing interest

The author has no competing interests to declare.
